# The Korean urban rural elderly cohort study: study design and protocol

**DOI:** 10.1186/1471-2318-14-33

**Published:** 2014-03-19

**Authors:** Eun Young Lee, Hyeon Chang Kim, Yumie Rhee, Yoosik Youm, Kyoung Min Kim, Ju Mi Lee, Dong Phil Choi, Young Mi Yun, Chang Oh Kim

**Affiliations:** 1 Department of Internal Medicine, Yonsei University College of Medicine, 50-1 Yonsei-ro, Seodaemun-gu, Seoul, Korea; 2 Department of Preventive Medicine, Yonsei University College of Medicine, 50-1 Yonsei-ro, Seodaemun-gu, Seoul, Korea; 3 Department of Sociology, Yonsei University, 50 Yonsei-ro, Seodaemun-gu, Seoul, Korea; 4 Department of Internal Medicine, Seoul National University Bundang Hospital, Seongnam, Korea

**Keywords:** Aging, Elderly, Cohort, Longitudinal study, Health

## Abstract

**Background:**

Korea is one of the fastest aging countries and is expected to become a super-aged society within 12 years. The Korean Urban Rural Elderly (KURE) study was developed to evaluate the epidemiological characteristics and establish the prevention and management of major disorders of the elderly in Korea.

**Methods/Design:**

The KURE study is a community-based prospective cohort study on health, aging, and common geriatric disorders of Korean elderly persons aged at least 65 years. To construct a cohort reflecting both urban and rural areas, we selected 2 representative communities in the country. To establish multidisciplinary approaches to geriatric health, this study was performed by researchers in the divisions of geriatrics, preventive medicine, endocrinology, and sociology. The baseline examinations began in 2012; the study will follow more than 4,000 elderly Koreans over 10 years. The first and second follow-up health examinations will be performed every 4 years. Every 2 years after each health examination, inter-assessment interview will be conducted to improve participant retention.

**Discussion:**

The KURE study will provide longitudinal epidemiologic data on health, aging, and common geriatric disorders of the elderly in Korea. This is a comprehensive, multidisciplinary study of the elderly with respect to biological, physical, socio-economic, and environmental factors. The results of this study will contribute to improve public health and welfare policies for the aging society in Korea.

## Background

Worldwide population aging has accelerated since the 1980s, with more than 11% of the world’s population already over the age of 60 years in 2010 [1]. The elderly population in Korea has increased with an unprecedented rate and is expected to be more than 20% of the total population in 2026, namely a super-aged society. This accelerated aging of the population may lead to a rapid increase in socioeconomic burden as well as medical costs. In Korea, the aging index ([population over the age of 65 years] ÷ [population under the age of 15 years] × 100) increased from 11.2% in 1980 to 68.4% in 2010. During the same period, the elderly dependency ratio ([population over the age of 65 years] ÷ [population aged 15–64 years] × 100) increased from 6.1% to 15.2%. The elderly dependency ratio is expected to be 20.0 in 2018, classifying as an aged society [2]. This suggests that the magnitude of the elderly population that depends on young adults will increase about 2-fold from 2000 to 2018.

In addition to aging itself, disability-adjusted life expectancy is also emerging as an important factor for public health in Korea. In 2005, disability-adjusted life expectancies in Korea were 67.5 years in men and 69.6 years in women, about 10 years shorter than the life expectancy in the same year. Many comorbidities occurring with aging have a great effect on disability-adjusted life expectancy. In addition, these comorbidities are characterized by multifaceted factors including biological, physical, psychosocial, and environmental factors. Therefore, we developed the Korean Urban Rural Elderly (KURE) study, a prospective longitudinal cohort of the elderly population in Korea, to obtain comprehensive data on health, aging, and prevalent disorders. The aim of this article is to describe the design of the KURE study.

## Methods/Designs

### Study design and participants

**Figure 1:**
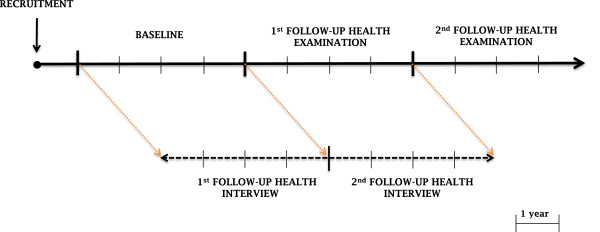
Conduct of the study.

This is a 12-year prospective cohort study of community-dwelling elderly persons aged 65 years and older living in urban or rural areas in Korea. To reflect the characteristics of the elderly residing in urban and rural areas, 3 administrative districts, called “Gu,” were selected as an urban area and one administrative district, called “Do,” was selected as a rural area. These areas were selected on the basis of their elderly populations and accessibility to the research center. Eligibility criteria, presented in Table 1, were chosen to maximize recruitment of community-dwelling elderly persons. Participants are mainly recruited by 1 of the 4 following methods: (1) recruiters visiting a random street and house, (2) participants visiting local government health facilities, (3) participants contact after seeing promotional poster for the study, or (4) acquaintances of study participants. During health examinations, participants undergo a thorough physical examination, face-to-face interview, and laboratory tests. Information gathered from interviews includes demographic, physical/clinical, nutrition, life style, psychological, daily activity, and social support and network. After the baseline health examination, participants will be taken full health examination every 4 years including face-to-face interview. Additional follow-up interviews will be performed between heath examinations (Figure 1). Participants will be followed for at least 10 years, or until death. To reduce losses to follow-up, a brief telephone interview is scheduled for the years when a full health examination is conducted and 2 years later. If initial contact by telephone fails, we will re-contact participants up to 5 times during different days and times of day. During the telephone interviews, information including change in residence, use of health services in the past year, and mortality is recorded. Furthermore, participants will be invited to free lectures on health and receive newsletters and holiday cards annually.

**Table 1:** Eligibility criteria for subject selection into the Korean urban rural elderly cohort study

**Inclusion criteria**	**Exclusion criteria**
• Age ≥ 65 years	• Age < 65 years
• Living in the study area: 3 northwestern districts of Seoul (Mapo-gu, Seodaemun-gu, and Eunpyeong-gu) and 1 rural area of Incheon (Ganghwa county)	• Living in the current residence less than 8 months of a calendar year
	• Planning to move from the current residence within 2 years
• Capable of communication with the research team	
• Provided written informed consent	

### Sample size calculations

To evaluate the relevance of the proposed sample size (n = 4,000), we estimated the minimum detectable odds ratio between an outcome and an exposure. We assumed the exposure frequency to be 25% and 33%, respectively. Prevalence of outcome (5-year mortality) was assumed with a range of 1%, 2%, 4%, and 16%. Power and a 2-sided alpha were set *a priori* at 90% and 0.05, respectively. Based on the 33% exposure frequency, the minimum detectable odds ratio was estimated to range from 2.33 for an outcome prevalence of 1% to 1.31 for an outcome prevalence of 16%. Based on the 25% of exposure frequency, the minimum detectable odds ratio was estimated to range from 2.44 for an outcome prevalence of 1% to 1.34 for an outcome prevalence of 16%.

### General health and medical history

Participants receive a comprehensive physical examination and complete a questionnaire by face-to-face interview (Table 2). All interviewers are trained for at least 2 days before administering the survey to participants. Medical information includes current and past medical history, family history, medication, subjective health status, vaccination, incontinence symptoms, falling, fracture, pain, and sleep. Chronic disease diagnoses by a physician are investigated by self-report using a list of 16 diseases including cardiovascular and respiratory disease, cerebrovascular attack, diabetes, thyroid disorders, arthritis, cataract, depression, and cancer. Consistent with the Korean National Health and Nutrition Examination Survey, all questionnaires about chronic disease consist of diagnosis time and process of treatment [3]. Smoking and alcohol consumption are also investigated using the questionnaire.

**Table 2:** Overview of the demographic and general health assessment in the Korean urban rural elderly cohort study

**Classification**	**Measures and instruments**
General health and medical history	Current and past medical history, family history, subjective health status, vaccination, incontinence symptoms, falling, fracture, pain, smoking, alcohol, sleep
Anthropometry	Height; weight; body mass index; circumference of waist, hip, thigh, and calf
Physical activities and function	Exercise (intensity and frequency), timed up and go test, grip strength, chair-rise test
Activities of daily living	Korean activities of daily living scale, Korean instrumental activities of daily living scale
Depression	Five Geriatric Depression Scale from the Korean version of comprehensive geriatric assessment, Korean version of the Geriatric Depression Scale - Short Form
Cognitive function	Korean version of Mini-Mental State Examination for dementia screening
Nutritional status and weight	Mini-nutrition assessment, loss of appetite, changes of body weight within the prior 3 months
Socioeconomic status	Education, occupation, household income, family income, marital status
Social support	Social network (size, range, homophily, density, degree centrality, closeness, betweenness, walk betweenness, brokerage, and overlapping), stressful life events

### Blood pressure and pulse rate

Blood pressure and pulse rate are measured using an electronic manometer (HEM-7111, Omron Healthcare Co., Ltd., Kyoto, Japan). Blood pressure and pulse rate are measured at heart-level with a standardized cuff. Values are recorded after 5 minutes of rest in the sitting position and repeated after another 5 minutes of rest. If blood pressure is significantly different between the first and the second measurements, a third measurement is taken after 5 minutes of rest. Radial arterial pulse waves are simultaneously measured noninvasively using an applanation tonometry and central blood pressure is calculated by the HEM-9000AI (Omron Healthcare Co., Ltd., Kyoto, Japan).

### Anthropometric measurements

During anthropometric measurements, all participants are asked to wear light clothing without shoes or jewelry. Height is measured within 0.1 cm using a stadiometer (DS-102, JENIX, Korea) with an upright position. Body weight is determined within 0.1 kg using an electronic scale (DB-150, CAS, Korea). Each device is calibrated prior to each examination. Body mass index (kg/m^2^) is calculated as weight in kilograms divided by the square of height in meters. Circumference of the waist, hip, thigh, and calf are measured within 0.1 cm using a plastic tapeline. Waist circumference is measured over the midpoint between the lower border of the ribs and iliac crest in the mid-axillary plane. Hip circumference is measured over the longest point of the buttocks. Thigh circumference is measured at the midpoint between the inguinal crease and proximal border of the patella. Calf circumference is measured at the largest point of the calf.

### Physical activities and function

Physical activity is assessed by a self-report scale: (1) non-active (mostly resting or lying down), (2) less active (mostly sitting without exercise), (3) active (mostly sitting but with regular light exercise, commuting, housework, standing at work, etc.), (4) very active (mostly standing or vigorous exercise). In addition, exercise is assessed according to the intensity, frequency, and average time.

The chair-rise test, a sit-to-stand test repeated 5 times, is performed by participants who are able to sit and stand. During the test, participants are instructed to do their best and the total time taken to complete the test is recorded in seconds. Timed up and go test is conducted on a leveled walking surface. At the beginning of the test, participants get up from a chair, walk 3 m at their regular walking speed, turn, walk back, and sit down again. Investigators record the total time from getting up to sitting down in seconds using a stopwatch. Grip strength is measured by a dynamometer in kilograms with participants’ arms resting on a desk with the elbow angled properly. Participants hold a dynamometer for as long as they can for a maximum of 3 seconds, and then relax their grip. This test is conducted in each of the hands and repeated after 20 seconds rest.

### Activity of daily living

Functional status of daily life is assessed using 2 types of questionnaires: the Korean version of activities of daily living [4] and a modified Korean version of instrumental activities of daily living [5-7]. The Korean version of activities of daily living is composed of 6 basic items such as bathing, dressing, feeding, rising or lying down, walking, and toileting. The modified Korean version of instrumental activities of daily living consists of 8 items such as shopping, using a phone, using public transportation, doing light house work, doing laundry, preparing meals, taking medicine, and managing money. The ability to perform each task is rated according to the degree of dependency on another person. These questionnaires validated among an elderly population [4,6].

### Depression and cognitive function

Depressive symptoms are assessed in 2 steps. First, participants are asked 5 questions about depressive symptoms using the Geriatric Depression Scale from Korean version of the comprehensive geriatric assessment [8]. The second step uses the Korean version of the Geriatric Depression Scale - Short Form [9,10], if participants answer “yes” to at least 1 of the 5 questions in the first step. The Korean version of the Geriatric Depression Scale - Short Form consists of 15 questions originating from the Geriatric Depression Scale with 30 items [11]; each question yields a score from 0 to 3 and the total score is used to evaluate depression as follows: depression (scores >10), moderate depressive symptom (scores from 6 to 9), and normal (scores from 0 to 5).

We administered the Korean version of the Mini-Mental State Examination for dementia screening to evaluate cognitive function and screen for dementia, which contains 30 items with scores ranging from 0 to 30 and includes areas of memory; attention; language; calculation; visuospatial abilities; and orientation to time, place, and person [12]. A total score of less than 24 indicates cognitive dysfunction. Recently, a total score of 27 has been suggested to detect mild cognitive impairment, especially among the elderly [13].

### Nutritional status and weight

Nutritional status is assessed by the Mini Nutritional Assessment, which consists of 6 questions on food intake and weight loss in the last 3 months, mobility, psychological stress or acute illness in the last 3 months, neuropsychological problems, and body mass index or calf circumference. Nutritional status was defined as follows: normal (scores from 12 to 14 points), at risk of malnutrition (scores from 8 to 11 points), and malnourished (scores from 0 to 7 points). Weight loss is classified as intended or unintended weight loss. Unintentional weight loss is related to a decrease in function and mobility in the elderly [14].

### Socioeconomic status and social support

Socioeconomic factors are assessed with regard to educational, occupational, financial, and marital status. Detailed data on socioeconomic status are composed of the highest level and duration of education, current and most long-term occupation, and subjective and objective financial status. Evaluation on social support is focused on social network as well as stressful life events. On the basis of the frequency, size, and closeness of social contact, social network is assessed to provide several parameters as follows: network size, range, homophily, density, degree of centrality, closeness, betweenness, walk betweenness, brokerage, and overlapping [15]. Participants are also asked whether they lived alone or with someone else (a spouse, children, or others) in the face-to-face interview. Previous studies have reported that social support was associated with beneficial effects on cardiovascular, endocrine, and immune systems and, furthermore, might regulate these physiologic responses in elderly [16].

### Blood and urine analysis

After an overnight fast, blood and urine samples are collected in the morning. Blood samples are centrifuged for 10 minutes at 3000 rpm 30 minutes after sampling. All samples are delivered to the Seoul Clinical Laboratories (Seoul, Korea) for analysis within 24 hours. As shown in Table 3, biochemical tests include complete blood count, kidney and liver function test, lipid profile, endocrine function, inflammatory markers, and urinalysis. Residual samples are stored at -80°C for future analysis.

**Table 3:** Laboratory and biochemical tests administered in the Korean urban rural elderly cohort study

**Classification**		**Measures**
Complete blood count		White blood cell, white blood cell differential, red blood cell, hemoglobin, hematocrit, mean corpuscular volume, mean corpuscular hemoglobin, mean corpuscular hemoglobin concentration, red cell distribution width, platelet count
Renal panel		Blood urea nitrogen, creatinine
Liver function test		Total protein, albumin, alkaline phosphatase, aspartate aminotransferase, alanine aminotransferase, total bilirubin
Lipid profile		Total cholesterol, triglyceride, high density lipoprotein cholesterol
Carotid ultrasonography		Carotid intima-media thickness, presence of plaque
Endocrine	Glucose metabolism	Glucose, glycated hemoglobin, insulin
	Bone metabolism	Calcium, phosphorus, parathyroid hormone, 25-hydroxyvitamin D, osteocalcin
	Thyroid function	Triiodothyronine, free thyroxine, thyroid stimulating hormone, thyroglobulin antibodies, thyroid peroxidase antibodies
Inflammation		Erythrocyte sedimentation rate, high sensitivity C-reactive protein
Others		Ferritin, uric acid
Urinalysis		Microalbumin, pH, nitrite, specific gravity, protein, glucose, ketone, bilirubin, blood, urobilinogen, red blood cells, white blood cells, epithelial cells, casts, bacteria, crystals, color
Bone mineral density		Dual energy X-ray absorptiometry
Electrocardiography		12-channel electrocardiography
Physical activity		Accelerometry
Body composition analysis		Bioimpedence test

### Bone mineral density and fracture risk assessment

The bone mineral density of the lumbar spine (L1–L4) and total hip are measured using dual-energy X-ray absorptiometry (Hologic QDR 4500A, Waltham, USA). The average precision error of this machine is less than 1.1%. All scans are performed by a trained operator on the same day as the health examination. Vertebral fracture assessment by dual-energy X-ray absorptiometry was performed to evaluate fracture in the spine. Fracture risk assessment was also calculated using the FRAX® model for South Korea [17].

### Carotid intima-media thickness (IMT) and plaque

Intima-media thickness (IMT) and plaques in both carotid arteries are assessed using high-resolution B-mode ultrasonography with an 8-MHz linear probe (L5-13IS, Samsung Medicine, Co., Ltd., Seoul, Korea). According to a predetermined, standardized protocol [18], carotid IMT is measured at the distal common carotid artery, 1 cm proximal to the carotid bulb, and the proximal internal carotid arteries from transverse and longitudinal orientations by an experienced technician. The IMT is defined as the distance between the adventitia-media interface and intima-lumen interface. The mean and maximum values of 6 measurements from the bilateral IMTs are used in the analyses. Carotid plaque is defined as the focal lesion protruding into the arterial lumen at least 0.5 mm or 50% greater than surrounding IMT value.

### Accelerometry

Accelerometers (ActiGraph GT3X-pus, ActiGraph Co., Pensacola, FL) are used to objectively measure physical activity and sleep time. Participants are instructed to wear the accelerometer on the wrist of their non-dominant arm for 7 consecutive days for 24 hours a day except during water activities, and to log wearing time. Physical activities are recorded using both 10- and 60-second intervals. At least 1 valid day of accelerometer wearing is to be included in the analysis, with a valid day defined as wearing the accelerometer for at least 10 hours in a 24-hour period. Accelerometry includes various parameters including step count, total activity counts, metabolic equivalent of task, and sleeping time. Accelerometers have been found useful for objectively measuring physical activity and to predict functional decline in community-dwelling elderly persons [19].

### Bioimpedence

To assess body composition, bioimpedence is measured using the InBody 720 (Biospace Co., Ltd., Seoul, Korea) with alternating currents of 250 mA and 6 frequencies (1 kHz, 5 kHz, 50 kHz, 250 kHz, 500 kHz, and 1 MHz) at 5 locations: right arm, left arm, trunk, right leg, and left leg. According to the manufacturer’s instructions, bioimpedence is measured in the morning and before eating, exercising, or bathing. With light cloths and no accessories, participants should stand barefoot on the foot electrodes and hold the hand electrodes. Arms and legs should be spread out so not to contact each other and other parts of body. Participants had to maintain this posture during the test. Bioimpedence analysis is a non-invasive, easy, and an inexpensive method for assessing body composition and is applicable to a wide range of subjects. In addition, bioimpedence has been shown to be a valid method for measuring body composition and visceral fat area in the elderly [20-22].

### Data collection and statistical analysis

All data are collected and managed using the database program (iCReaT, Korea National Institutes of Health, Korea Centers for Disease Control and Prevention). Data are verified by double checking for erroneous or missing values. Only trained and authorized persons are able to access the database system. All participants are interviewed with structured questionnaires, which are designed for this study. Interviewers record responses on paper forms; all information is kept in a locked space. Secondary data will be collected from national registries to obtain information on the use of health services and mortality. Trained health investigators and medical laboratory technicians perform physical examination and laboratory tests, respectively. They are instructed to follow the standardized clinical protocol.

Data will be presented as mean ± standard deviations, percentages, odds rations (for cross-sectional analysis) or relative risks (for longitudinal analysis). The prevalence of various geriatric disease and related-factors will be estimated according to age, gender, and areas. Outcomes will include mortality, newly-developed diseases or disabilities and any other health issues related with the elderly. Student t-test or analysis of variance will be used to compare variables among the different groups. To investigate the associations between outcomes and studied parameters, multivariate analysis will be used. Possible confounding factors, which are found in univariate analysis, as well as clinically relevant parameters will be adjusted in multivariate analysis. The level of P value less than 0.05 will be considered as statistical significance.

### Ethics

The study protocol was approved by the ethics committee of Severance Hospital. Participants provided written informed consent (including permission to use secondary data such as register data on health service use) before enrolling in the study and completing the first examination. Participants were informed that they could chose to withdraw from the study at any time. Interviews were performed in a separate room to protect participants’ privacy. According to the International Conference on Harmonization Good Clinical Practice guidelines, all information recorded was anonymized.

## Discussion

This study protocol describes the design and methods used in the KURE study. The KURE cohort study is designed to assess health status, aging, and major geriatric diseases in community-dwelling elderly persons in Korea. In this study, we focus on not only epidemiologic characteristics of geriatric disorders, but also the relationship between social support (network) and geriatric health in terms of cardiovascular, neuroendocrine, and immune system. Hence, this study is able to evaluate the impact of the social environment of the elderly on their health and quality of life in addition to elucidating biological risk factors of geriatric disease.

There are several strengths in this study. First, this is the cohort study for long follow-up time. Therefore, this study may assist in determining causal associations between predicted determinants of health and outcomes and be used to obtain trajectories of geriatric health over time [23,24]. Second, this study has a large sample size, with a statistical power to detect mild to moderate associations. Third, the face-to-face interview method may be especially useful in obtaining accurate information, especially among the elderly who may not immediately understand or response the questionnaire. Fourth, this study recruited the elderly from both urban and rural areas. This will help us investigate geriatric health in different geographic and socioeconomic environments and life styles. Lastly, the KURE investigative team is multidisciplinary including experts in geriatrics, preventive medicine, infection and immunology, endocrinology, and sociology. This team approach may be helpful in developing a unique research resource for a multifaceted understanding of geriatric health. The major limitation of this study is to recruit only voluntary participants. This might cause selection bias and not completely represent the whole geriatric population in Korea.

The KURE study will contribute to establishing how various factors such as biological, physical, socio-economical, and environmental factors affect geriatric health and disease by providing comprehensive epidemiologic data of geriatric health in Korea. This will not only improve the understanding of disease prevalence in the elderly, but also assist in preventing disease, and ultimately improving the health management system in Korea.

## Abbreviations

IMT: Intima media thickness; KURE: Korean urban rural elderly.

## Competing interests

The authors declare that they have no competing interests.

## Authors’ contributions

EYL contributed to the drafting and revision of the manuscript, supervision of the study, and acquisition of data. HCK contributed to the revision of the manuscript, design/supervision of the study, and statistical methodology. YR contributed to the revision of the manuscript and design/supervision of the study. YY contributed to the revision of the manuscript and design/ supervision of the study with specific focus on social network. KMK contributed to the revision of the manuscript and supervision of the study. JML contributed to the revision of the manuscript, design/supervision of the study, and acquisition of data. DPC contributed to the revision of the manuscript, supervision of the study, and acquisition of data. YMY contributed to the revision of the manuscript and acquisition/management of data. COK contributed to the revision of the manuscript and design/supervision of the study. All authors read and approved the final manuscript.

## Pre-publication history

The pre-publication history for this paper can be accessed here:

http://www.biomedcentral.com/1471-2318/14/33/prepub
